# Measurement of three-dimensional morphological characteristics of the calcaneus using CT image post-processing

**DOI:** 10.1186/1757-1146-7-19

**Published:** 2014-03-14

**Authors:** Minfei Qiang, Yanxi Chen, Kun Zhang, Haobo Li, Hao Dai

**Affiliations:** 1 Department of Orthopedic Trauma, East Hospital, Tongji University School of Medicine, 150 Jimo Road, 200120 Shanghai, China

**Keywords:** Three-dimensional measurement, Calcaneus, Computed tomography, Computer-assisted, Image processing

## Abstract

**Background:**

Although computed tomography (CT) with three-dimensional (3D) rendering of the calcaneus is used for diagnostic evaluation of disorders, morphological measurements for the calcaneus are mostly based on a two-dimensional plane. The purposes of this study were to design a method for 3D morphological measurements of the normal calcaneus based on CT post-processing techniques, to measure morphological parameters in the male and female groups and describe gender differences of the parameters, and to investigate the reliability of such measurements.

**Methods:**

One hundred and seventy-nine patients (83 men and 96 women) with a mean age of 40.6 (range, 21 to 59) years who underwent CT scans for their feet were reviewed retrospectively. The 3D structure of a normal calcaneus after shaded surface display reconstruction was extracted by interactive and automatic segmentation. Morphological measurements were achieved by means of a 3D measurement method based on CT image post-processing. Lengths and heights of the main parts of the calcaneus, Gissane’s angle, Böhler’s angle and the area of articular facet were worked out in 3D space. Gender-related size differences of parameters were compared using analysis of covariance (ANCOVA), adjusting for body height. Intra-observer and inter-observer reliabilities were assessed using intraclass correlation coefficients (ICCs) and the root mean square standard deviation (RMS-SD) for precision study.

**Results:**

A large range of measurement values was found. Only the length of the anterior process was without gender difference (*p* > 0.05). The other parameters in the male group were greater than those in the female group (*p* < 0.01 for each, ANCOVA). All parameters had excellent reliability and reproducibility (ICC > 0.8). Precision was acceptable for intra-observer RMS-SD (linear, angular and areal measurements no more than 0.6 mm, 1.2° and 0.25 mm^2^, respectively). Inter-observer RMS-SD ranged from 0.4 to 1.6 mm for linear measurements, 1.2 to 2.5° for angles and 0.24 to 0.40 mm^2^ for areas.

**Conclusions:**

Three-dimensional morphological measurement based on a CT post-processing technique was highly reliable and repeatable for calcaneal anatomic morphological measurement. The current data will be helpful for anatomic reduction of calcaneal fractures and calcaneal malunion.

## Background

The calcaneus is the most commonly fractured tarsal bone. Calcaneal fractures account for about 2% of all fractures and 60% of tarsal bone fractures. Roughly 75% of calcaneal fractures are intra-articular [1,2]. Studies have suggested that the anatomic reduction of calcaneal fractures is important for feet and ankles in functional outcomes, and that the purpose of the treatment for calcaneal fractures is to restore the height, length, width and axis of the calcaneus and articular facet congruency [3-7]. The arrival of computed tomography (CT) revolutionized the anatomic assessment of calcaneal fractures and improved therapeutic decision-making [8]. Coronal and axial CT scans were also used to measure and define some calcaneal displacement parameters, which provided a quantitative assessment of calcaneal fractures and improvement achieved with open reduction [9]. Lateral radiographs were used to measure calcaneal parameters including angles and distances, and those relevant reference values were summarized and described in detail in a previous study [10].

Most morphological measurements about the calcaneus were based on the two-dimensional (2D) plane. The main methods included the following: anatomical measurement of the cadaver; measurement based on X-rays; CT scans, and 3D reconstruction images based on a hospital graphics workstation. The above methods have the following implications: Firstly, despite results being accurate, cadaver specimens may be hard to obtain. Clinicians may also have difficulty in carrying out a large-scale prospective study. Secondly, radiographs may be affected by the orientation of the patient’s foot and the difference among casting degrees of the tube because of foot pain and swelling in the acute injury [11]. Finally, the Picture Archiving and Communication System (PACS) in the hospital usually outputs the 2D projection images of 3D reconstruction graphics, which can be only browsed with a fixed view by clinicians, and thus the measurement remains 2D. Recently, many techniques for 3D bone measurements have become available, which allow surface, distance and angular measurements on models generated from CT or magnetic resonance imaging [12-15]. However, little data is currently available about measuring the 3D morphological parameters of the calcaneus in a large sample.

Studies involving quantitative parameters need scientific and accurate measurement and evaluation methodology [13]. Computer-assisted technology, such as preoperative planning and surgical navigation, has been used widely in the surgical management [16]. Image analysis and processing, as one of the computer-assisted technologies, involves generating 3D models and obtaining relevant information from the data. The hypothesis of the current study is that a reliable and repeatable method for 3D morphological measurements of the calcaneus based on a CT post-processing technique is designed. Clinically relevant 3D morphological parameters of the normal calcaneus will be obtained in the male and female groups respectively and serve as control data for calcaneal disorders. In addition, gender differences of the parameters will be detected as well.

## Methods

### Study subjects

From December 2009 to January 2012, 335 cases from the Department of Foot and Ankle underwent CT scans. The CT scans of 135 cases were excluded because of calcaneal fractures, deformity of feet, osteoarthritis or bone tumors. Two hundred cases encountered fracture of the talus, ankle and other related structural injuries. Among the 200 cases, CT scans were performed twice in 9 patients and three times in 6 patients. Patients with CT records on more than one occasion were included only once. Therefore, 179 consecutive adult, normal, calcaneal, CT data were retrospectively analyzed in the study. The included patients consisted of 83 men (46.4%) and 96 women (53.6%) with a mean age of 40.6 (range, 21 to 59) years. The main general demographic data of the included subjects were reported in Table 1. This study was approved by the institutional review board of the hospital (The Committee of the Medical Ethics, East Hospital of Tongji University in Shanghai, China) and informed consents were obtained from all participants.

**Table 1:** General demographic data of the subjects

	**All (n = 179)**			**Male (n = 83)**			**Female (n = 96)**			***p *** **Value**
	**Mean (SD)**	**Min**	**Max**	**Mean (SD)**	**Min**	**Max**	**Mean (SD)**	**Min**	**Max**	
Age (year)	40.6 (10.2)	21	59	39.6 (9.8)	22	55	41.5 (10.5)	21	59	0.214
Weight (kg)	63.5 (11.8)	43.0	92.5	75.2 (5.0)	62.5	92.5	53.5 (4.5)	43.0	63.5	< 0.01
Height (m)	1.67(0.07)	1.55	1.81	1.74 (0.05)	1.63	1.81	1.61 (0.04)	1.55	1.68	< 0.01
BMI (kg/m^2^)	22.5 (2.7)	17.3	29.1	25.0 (1.4)	20.4	29.1	20.5 ( 1.5)	17.3	24.0	< 0.01

BMI = Body mass index.

### CT scans and image post-processing

**Figure 1:**
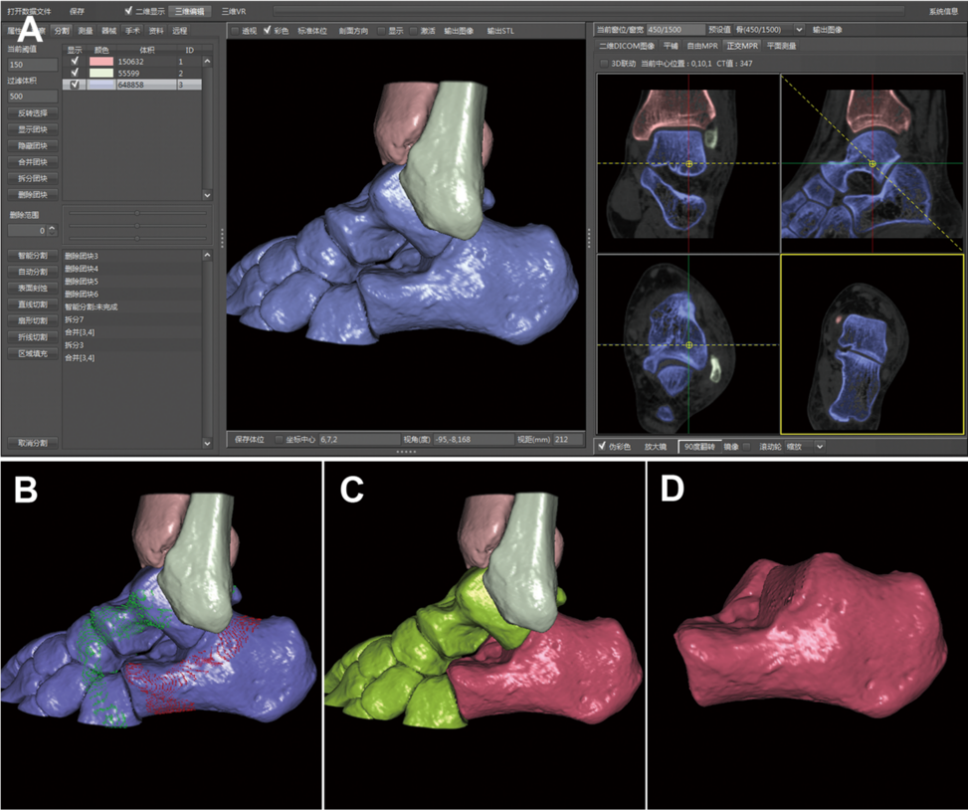
**Process of generating the 3D structures of the calcaneus. (A)** Joints of the hindfoot containing the subtalar joint, calcaneocuboid joint and talonavicular joint combined with each other, which showed the blue clumps. These bones couldn’t be recognized automatically by surface rendering in 3D Imaging. **(B)** The calcaneus and other bones were labeled separately by 3D interactive and automatic segmentation. After the Enter key was clicked to start the computer processing, the color of labeled bones gradually turned red and green and then the division was finished automatically in one minute. **(C)** The calcaneus was marked red and others were marked green at last. **(D)** The calcaneus was extracted and other bones were deleted.

All data were collected from the Department of Radiology, which were saved in the DICOM 3.0 format (.dcm). CT was performed using a 16–detector row CT scanner (GE Light-Speed CT; Waukesha, WI, USA). Imaging parameters for CT scanning were as follows: section thickness, 0.625 mm; tube voltage, 120 kVp; pitch, 1.375; matrix, 512 × 512. Thin-slice CT transverse images of all subjects were firstly uploaded to the PACS, and then these CT data were input into a computer-aided orthopedic clinical research platform (SuperImage orthopedics edition 1.0, Cybermed Ltd, Shanghai, China) via removable storage devices [13,17]. The bone and non-bone materials were defined via assigning CT density threshold (of Hounsfield units). The 3D structures of each calcaneus and its surrounding bones were reconstructed by shaded surface display (SSD) with a reconstruction interval of 0.625 mm and the density threshold of 150H. A 3D interactive and automatic segmentation technique was applied to distinguish all component bones. The bones of the forefoot, distal tibia, talus, navicular bone, cuboid bone, and cuneiform bones were removed manually for the purpose of 3D measurements. Then, the calcaneus was generated and observed (Figure 1). Morphological parameters of the calcaneus were measured according to combined measurement techniques including essential elements of a point, line and surface in 3D space. The calcaneus could be rotated with any viewing angle while being measured in a user interface as shown in an additional movie file for more detail (see Additional file 1).

### The 3D measurements of calcaneal morphological parameters

**Figure 2:**
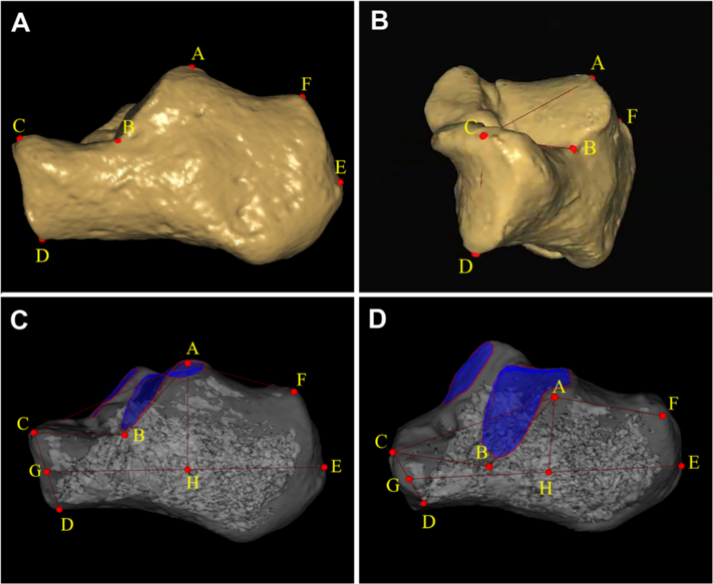
**Three-dimensional morphological measurements of calcaneus. (A, B)** Location of measurement points was illustrated. *A* = highest point of posterior facet; *B* = bottom of the posterior facet at lateral surface intersecting anterior process of calcaneus; *C* = highest point of calcaneocuboid joint; *D* = lowest point of calcaneocuboid joint; *E* = most posterior point of calcaneal tuberosity; *F* = highest point on the superior edge of calcaneal tuberosity. **(C, D)** The calcaneal morphological parameters in 3D space were shown. *GE* = length of calcaneal axis; *AH* = height of posterior facet; *AB* = length of posterior facet; *BC* = length of anterior process; *CD* = height of anterior process; *∠ABC* = Gissane’s angle; 180°-*∠CAF* = Böhler’s angle. The posterior and middle facets were shown as blue surfaces.

The calcaneal anatomic landmarks on bone surface meshes were selected including: the highest point of the posterior articular facet (point A), the bottom of the posterior articular facet at the lateral surface intersecting the anterior process of the calcaneus (point B), the highest point of the calcaneocuboid joint (point C), the lowest point of the calcaneocuboid joint (point D), the most posterior point of the calcaneal tuberosity (point E), and the highest point on the superior edge of the calcaneal tuberosity (point F). Point H was the foot of the perpendicular segments AH on the calcaneal axis (Figure 2).

After all of the above landmarks were assessed and defined, 3D measurement was as follows (Figure 2):

● The length of the calcaneal axis (LCA): the distance from point G (the midpoint between C and D) to point E.

● The height of the posterior facet (HPF): the perpendicular distance from point A to the calcaneal axis.

● The length of the posterior facet (LPF): the distance between A and B.

● The length of the anterior process (LAP): the distance between B and C.

● The height of the anterior process (HAP): the distance between C and D.

● The critical angle of Gissane (GA): the intersection of line AB and line BC.

● The tuber angle of Böhler (BA): the intersection of line AC and line AF.

● The areas of the posterior subtalar joint (APF) facet and the middle facet (AMF) were shown as blue surfaces (Figure 2D).

**Figure 3:**
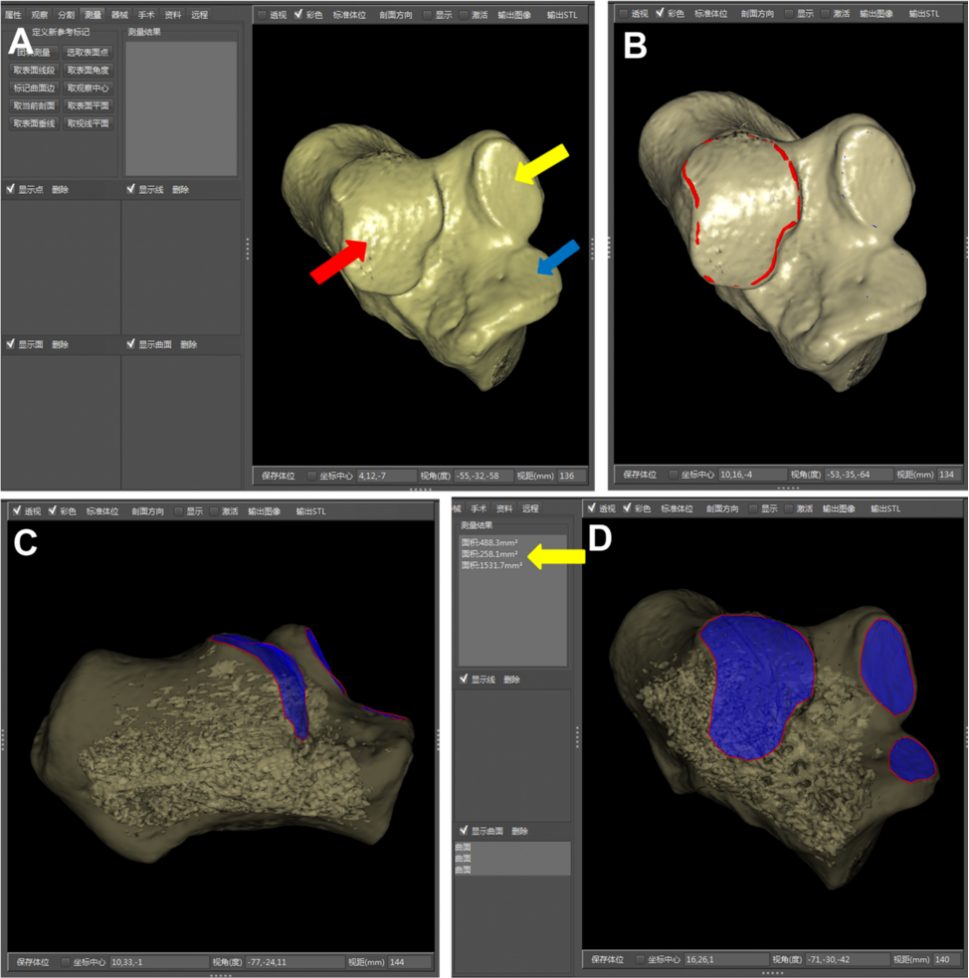
**Areal measurements of subtalar articular facets for example. (A)** After the segmentation, the SSD reconstruction image of the 3D calcaneus presented the anterior (blue arrow), middle (yellow arrow) and posterior (red arrow) facets clearly. **(B)** The operator marked the border of the articular facet. The border (red curve) was actually unbroken, though part of the red curve seemed not visualized. **(C)** With the help of the perspective mode of SSD reconstruction, the defined boundary of the surface could be observed. **(D)** On the anterolateral view, the boundary of each articular facet was clear. Each area was calculated by the software (yellow arrow).

Before areal measurement was calculated by the computer software, the operator needed to mark the border of articular facets at first (Figure 3). With the help of the perspective mode of SSD reconstruction, the defined boundary of the surface could be observed clearly (Figure 3C, D).

### Reliability assessment

The intraclass correlation coefficient (ICC) for reliability was used to test at a desired lower limit of 0.8 and a 95% confidence interval (CI) of 0.2. According to Bonett’s approximation, at least 36 patients were required based on reliability testing [18]. Thirty-six subjects were randomly selected to assess the intraobserver reproducibility and interobserver reliability. Three surgeons took the measurement independently. Before they took measurements, the measuring technique and calcaneal anatomic landmarks were standardized. The main examiner finished all the measurements of 179 subjects. The 36 subjects were measured again by the main examiner in different sessions, and one time separately by the other examiners. The average ICC of inter-observer reliability was calculated from all obtained measurements among three examiners. The root mean square standard deviation (RMS-SD) was calculated for the measurement precision.

### Statistical analysis

The data were analyzed using SPSS 18.0 software for Windows (Chicago, IL, USA). We analyzed the intra-observer and inter-observer reliability using ICCs. 95% CIs were determined in the setting of a single measurement and absolute agreement, as a two-way random model was selected. The data were represented with mean ± standard deviation (SD). Differences in general demographic data between groups were tested using the independent samples *t* test. Morphological parameters assessed by 3D measurements were compared between male and female groups using analysis of covariance (ANCOVA), adjusting for body height. A *p* < 0.05 was considered statistically significant. The RMS-SD was used to assess 3D angular precision of foot bones from quantitative CT at the level of the individual participant [12]. The unit of the RMS-SD was the same to the parameter. The equation for calculating the RMS-SD:

$$\mathrm{RMS}-\mathrm{SD}=\sqrt{\frac{\sum SD^{2}}{N}}$$

In the equation, the SD was calculated by repeating measuring each parameter for each of the 36 feet. SD values in intra-observer precision represented variability when the same examiner repeated measurements. SD values in inter-observer precision represented variability across two examiners. For linear and areal measurements, the coefficient of variation (CV) was defined as the ratio of the measurement precision to the mean value.

## Results

Table 2 summarized the mean value, SD, minimum and maximum values of the calcaneal morphological parameters over all subjects and also within the two gender groups separately. ANCOVA with body height as the covariate was used to investigate possible gender differences of the morphological parameters. The average LCA, HPF, LPF and HAP in the male group were significantly relatively greater than those in the female group (*p* < 0.01 for each). However, there was no significant gender-related size difference in LAP (*p* > 0.05). The average Gissane’s angle was 127.7° ( range, 113° to 144°) in the male group and 123.5° (range, 110° to 135°) in the female group. Male Böhler’s angle ranged from 33° to 45° with the mean of 39.6°. Female Böhler’s angle ranged from 23° to 39° with the mean of 30.7°. The gender-related differences of the two angles were statistically significant (*p* < 0.01). The average APF and AMF in the male group were 14.5 cm^2^ and 6.1 cm^2^ and those in the female group were 12.9 cm^2^ and 5.2 cm^2^, respectively. Significant differences in areas were observed between two groups (*p* < 0.01).

**Table 2:** Three-dimensional morphological parameters of the normal calcaneus

	**All (n = 179)**			**Male (n = 83)**			**Female (n = 96)**			***p *** Value^*^
	**Mean ± SD**	**Min**	**Max**	**Mean ± SD**	**Min**	**Max**	**Mean ± SD**	**Min**	**Max**	
LCA (mm)	74.2 ± 6.0	64.2	85.9	80.0 ± 3.4	71.3	85.9	69.2 ± 1.9	64.2	74.3	<0.001
HPF (mm)	28.5 ± 3.2	23.8	35.1	31.5 ± 1.8	28.5	35.1	25.9 ± 1.1	23.8	28.2	<0.001
LPF (mm)	25.3 ± 3.9	19.5	34.5	28.6 ± 2.9	24.3	34.5	22.4 ± 1.8	19.5	27.2	<0.001
LAP (mm)	21.0 ± 1.6	17.5	24.2	21.1 ± 1.6	18.5	24.2	20.8 ± 1.5	17.5	23.5	0.374
HAP (mm)	27.2 ± 3.5	22.4	33.6	30.4 ± 1.8	27.2	33.6	24.4 ± 1.7	22.4	29.8	<0.001
GA (deg)	125.4 ± 6.2	110	144	127.7 ± 6.4	113	144	123.5 ± 5.4	110	135	<0.001
BA (deg)	34.8 ± 5.3	23	45	39.6 ± 2.6	33	45	30.7 ± 3.2	23	39	<0.001
APF (cm^2^)	13.6 ± 1.2	11.1	16.2	14.5 ± 1.0	11.9	16.2	12.9 ± 1.0	11.1	14.6	<0.001
AMF (cm^2^)	5.6 ± 0.6	4.7	7.4	6.1 ± 0.5	5.5	7.4	5.2 ± 0.3	4.7	6.5	<0.001

^*^P values for gender differences were from ANCOVA using body height as the covariate.

LCA = length of calcaneal axis; HPF = height of posterior facet; LPF = length of posterior facet; LAP = length of anterior process; HAP = height of anterior process; GA = Gissane's angle; BA = Böhler's angle; APF = area of posterior facet; AMF = area of middle facet.

**Figure 4:**
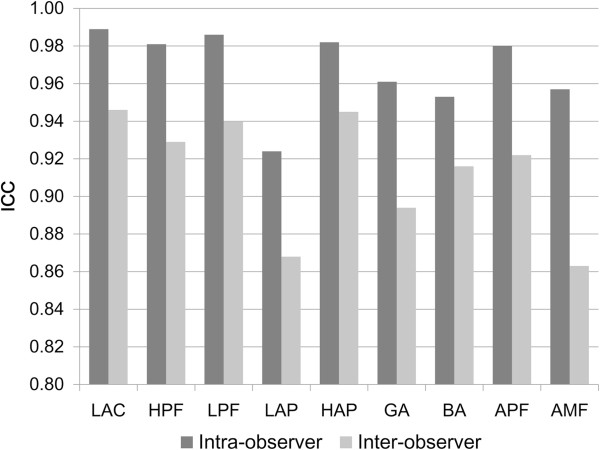
**Intra-observer and inter-observer reliabilities of 3D morphological measurements (n = 36).** The average ICC of inter-observer reliability was calculated from all obtained measurements among three examiners. All the parameters exhibited high ICC values, including length of calcaneal axis (LCA), height of posterior facet (HPF), length of posterior facet (LPF), length of anterior process (LAP), height of anterior process (HAP), Gissane's angle (GA), Böhler's angle (BA), area of posterior facet (APF), and area of middle facet (AMF).

The ICC values of intra-observer and inter-observer reliabilities of all parameters were revealed (Figure 4). The 3D morphological measurement for the normal calcaneus was reliable with the intra-observer reproducibility coefficients of 0.924 or greater. In inter-observer reliability among three examiners, average ICC values of every parameter were more than 0.8. Table 3 revealed the RMS-SD for intra-observer and inter-observer precision of 3D calcaneal morphological measurements, as well as the CV. The intra-observer RMS-SD values of linear, angular and areal measurements were no more than 0.6 mm, 1.2° and 0.25 mm^2^, respectively. The inter-observer RMS-SD ranged from 0.4 to 1.6 mm for linear measurements, 1.2 to 2.5° for angles and 0.24 to 0.40 mm^2^ for areas.

**Table 3:** Intra- and inter-observer precision of 3D calcaneal morphological measurements

	**Intra-observer**		**Inter-observer**					
	**Examiner 1**		**Examiner 1 vs. 2**		**Examiner 1 vs. 3**		**Examiner 2 vs. 3**	
	**RMS-SD**	**CV (%)**	**RMS-SD**	**CV (%)**	**RMS-SD**	**CV (%)**	**RMS-SD**	**CV (%)**
LCA (mm)	0.6	0.8	1.5	2.1	0.8	1.0	1.6	2.2
HPF (mm)	0.4	1.6	0.5	1.9	1.1	3.8	1.1	3.8
LPF (mm)	0.5	1.9	0.7	2.9	1.0	4.1	1.3	5.1
LAP (mm)	0.4	1.9	0.6	3.0	0.4	2.1	0.6	2.7
HAP (mm)	0.4	1.7	0.6	2.3	0.8	3.0	0.8	3.2
GA (deg)	1.2	NA	2.2	NA	1.6	NA	2.5	NA
BA (deg)	1.0	NA	1.2	NA	1.5	NA	1.7	NA
APF (cm^2^)	0.25	1.9	0.40	3.0	0.31	2.3	0.37	2.8
AMF (cm^2^)	0.19	3.6	0.24	4.5	0.28	5.1	0.34	6.2

RMS-SD = root mean square standard deviation; CV = coefficient of variation; NA = not applicable; LCA = length of calcaneal axis; HPF = height of posterior facet; LPF = length of posterior facet; LAP = length of anterior process; HAP = height of anterior process; GA = Gissane's angle; BA = Böhler's angle; APF = area of posterior facet; AMF = area of middle facet.

## Discussion

The calcaneus, the largest tarsal bone, is an irregular shaped bone with complex contour and articular facets. The integrity of calcaneal anatomic morphology is of important clinical value to maintain normal function of the hindfoot and supporting modality of the arch, and to ensure stress conduction for weight bearing [5-7,19,20]. To assess the reduction for calcaneal fractures, comparing preoperative and postoperative radiographs was a common method [21]. The calcaneal morphological parameters included relevant angles, distances and areas. Sometimes, the standard anteroposterior, lateral, and axial radiographs are quite difficult to obtain in acute injury because of foot pain and swelling. Comparing preoperative and postoperative radiographs for anatomical reduction would be seriously affected. Most calcaneal fractures are intra-articular. CT scans were suggested to be the routine examination for intra-articular injuries [3,22,23]. Three-dimensional CT images would be helpful for assessment of intra-articular fractures.

Gissane’s and Böhler’s angles were the commonly assessed angles in calcaneal fractures. They could give information on the severity of the injury. Especially, the restoration of Böhler’s angle is an important prognostic factor that is correlated with outcomes [5,20]. Gissane’s angle was formed by the posterior facet and anterior facet and the normal value was between 120° and 145° [1]. Restoring the relationship of the articular surfaces required the reconstruction of Gissane’s angle. Böhler’s angle was formed by the superior edges of the tuberosity, the subtalar joint and the anterior process and it normally ranged from 25° to 40° on lateral radiographs [21]. In this study, four landmarks (point A, B, C and F) decided the two angles in 3D space. The results of the two angles were not consistent with those available in the literature [1,21]. Moreover, both angles distinguished between genders. Generally, Böhler's angle decreased or even reversed and Gissane’s angle increased in the fracture patterns where the whole posterior facet collapsed. If only the lateral portion of the posterior facet is involved, Gissane’s angle may remain normal or not significantly increase [24]. The loss of calcaneal height could cause a more horizontal talus (loss of the talar declination angle) and anterior tibiotalar impingement, and a decrease of ankle dorsiflexion [25]. The valgus or varus deformity of the calcaneus may occur, and the unsatisfactory reduction may result in a severely disabling fracture malunion.

The reduction of the anterior process followed by the posterior facet allows fracture fragments to be easily manipulated into an anatomic position via the extensile lateral approach [26]. Reduction of the anterior process distal to Gissane’s angle is important in restoring lateral column length. The calcaneal axial length sometimes shortens in the intra-articular calcaneal fractures or the compression type of anterior process fractures. For simple injury patterns such as Sanders type IIA and IIB fractures, percutaneous reduction and screw fixation is an optional method [6]. One or two screws (axial screws) are directed from the posterior of tuberosity (near point E) into the dorsal portion of the anterior process. Therefore, the calcaneal axial length is not only helpful for diagnosis, but also for deciding the size of axial screws during surgical procedures. Gupta et al. reported that the axial calcaneal length (axial CT scan) was measured from the tip of the posterior tuberosity to the most distal calcaneocuboid joint surface and that postoperative average value was 77 mm [9].

When the talocalcaneal angle was measured on the lateral radiograph, either the center line of the calcaneus or the tangent to the bottom of the calcaneus was used to present the calcaneal axis [10,27]. The calcaneal axis also involves the height of the posterior facet. The height of the posterior facet was the perpendicular distance from the highest point of the posterior facet to the calcaneal axis. The calcaneal axial based on 3D measurement was similar to the centerline of the calcaneus (Figure 2). The value of HPF was quite different from that mentioned in the literature because of the two different calcaneal axes selected [28]. Leung et al. adopted the tangent to the bottom of the calcaneus as the calcaneal axis, and reported that the mean of the normal height of the posterior facet was 49 mm on lateral radiographs [28].

The anterior and middle facets in some cases were discrete and some were combined [29,30]. When the articular facets were conjoined, we measured the part of area on the sustentaculum tali as the area of middle facet, and the area on the calcaneal beak was eliminated. In the past, measuring the area of the curved surface in cadavers directly using measuring tools may be quite complex. With the help of surface measurements based on the CT post-processing technique, it was convenient to observe and measure the area of articular facets. The results may provide a group of reference data to confirm further the relationship between the quality of reduction of the subtalar joint facet and the prognosis of calcaneal fractures. Some studies have shown that even step-offs in the joint surface of 1–2 mm would lead to a load redistribution within the posterior joint facet of the calcaneus, inferior functional scores, and a higher rate of posttraumatic subtalar arthritis [3,31-33]. The quality of reduction of the subtalar joint facet, especially the posterior facet after operative management, was related to outcome of calcaneal fractures. Concerning clinical relevance, those reference data in the present study need to be validated in further clinical studies.

The advantage of 3D measurements is not defining some concept but reproducibility. With the development of computer science, basic theories and methods for CT post-processing and image processing algorithms are widely used. Many software platforms can also perform accurate 3D measurements on a patient’s anatomy [12,14,34]. Gutekunst et al. developed the methods to define bone axes using landmarks on quantitative CT bone surface meshes to assess 3D foot bone-to-bone orientation angles, which presented the angular measurement with moderate to high ICC values and high precision [12]. The present study also indicated that the linear, angular and areal measurements exhibited high ICC values and acceptable precision. The repeatability of measurements was carried out by three examiners with excellent inter-observer reliability. When the reliable landmarks are defined in 3D space, the distance and angles deduced from those landmarks will be reliable. Three-dimensional SSD as one research focus involves a computer visual and pattern recognition technique. If more CT imaging reprocessing workstations could implant the SSD technique, original data from a radiological database would be explored more effectively. The research for skeletal morphology would be widely carried out and tend towards accurate quantization.

Several limitations were noted in this study. Firstly, CT data of bilateral foot were not available. Considering the additional cost and patients’ concerns about the extra dose of radiation, patients were not asked to receive more than one CT scan. Thus, the correlation of right to left for these parameters was not analyzed. Secondly, the foot plane was hard to confirm from CT scans, so the calcaneal inclination angle was not measured, which was formed by the line connecting the most inferior surface of the tuberosity and the foot plane. Thirdly, the width of the calcaneus was not measured, either. The width cannot be described clearly by two constant landmark points, for the calcaneus is an irregularly shaped bone. This study did not describe the lateral talocalcaneal angle and the tibiocalcaneal angle, which involve the talus and the tibia. Other bones around the calcaneus need to be taken into account in future studies.

## Conclusions

This study demonstrates that the 3D morphological measurement based on a CT image post-processing technique is highly reliable and repeatable. The current data will be helpful for anatomic reduction of calcaneal fractures and calcaneal malunion. With the development of CT reprocessing, it will be easy to obtain anatomic parameters of bones in large samples. The clinical value of 3D measurements for the calcaneus will be further investigated in future prospective clinical studies.

## Abbreviations

CT: Computed tomography; 3D: Three-dimensional; 2D: Two-dimensional; PACS: Picture archiving and communication system; SSD: Shaded surface display; LCA: Length of calcaneal axis; HPF: Height of posterior facet; LPF: Length of posterior facet; LAP: Length of anterior process; HAP: Height of anterior process; GA: Gissane's angle; BA: Böhler's angle; APF: Area of posterior facet; AMF: Area of middle facet; ICC: Intraclass correlation coefficient; CI: Confidence interval; RMS-SD: Root mean square standard deviation; CV: Coefficient of variation.

## Competing interests

The authors declare that they have no competing interests.

## Authors’ contributions

Each Author has contributed substantially to study design, literature research, data acquisition, data analysis, and manuscript drafting. All authors have read and approved the final manuscript.

## Supplementary Material

Additional file 1 **The calcaneus could be rotated with any viewing angle while being measured in a user interface.** The anatomic landmarks were shown in various views including lateral view, anterior view, superior view and so on. All distances, angles and area were measured in 3D space. Click here for file
